# Behavioral and Neural Markers of Flexible Attention over Working Memory in Aging

**DOI:** 10.1093/cercor/bhw011

**Published:** 2016-02-09

**Authors:** Robert M. Mok, Nicholas E. Myers, George Wallis, Anna Christina Nobre

**Affiliations:** Department of Experimental Psychology and Oxford Centre for Human Brain Activity, University of Oxford, Oxford OX3 7JX, UK

**Keywords:** alpha, healthy aging, magnetoencephalography, oscillations, working memory

## Abstract

Working memory (WM) declines as we age and, because of its fundamental role in higher order cognition, this can have highly deleterious effects in daily life. We investigated whether older individuals benefit from flexible orienting of attention within WM to mitigate cognitive decline. We measured magnetoencephalography (MEG) in older adults performing a WM precision task with cues during the maintenance period that retroactively predicted the location of the relevant items for performance (retro-cues). WM performance of older adults significantly benefitted from retro-cues. Whereas WM maintenance declined with age, retro-cues conferred strong attentional benefits. A model-based analysis revealed an increase in the probability of recalling the target, a lowered probability of retrieving incorrect items or guessing, and an improvement in memory precision. MEG recordings showed that retro-cues induced a transient lateralization of alpha (8–14 Hz) and beta (15–30 Hz) oscillatory power. Interestingly, shorter durations of alpha/beta lateralization following retro-cues predicted larger cueing benefits, reinforcing recent ideas about the dynamic nature of access to WM representations. Our results suggest that older adults retain flexible control over WM, but individual differences in control correspond to differences in neural dynamics, possibly reflecting the degree of preservation of control in healthy aging.

## Introduction

Working memory (WM) is essential for much of higher order cognition. By enabling us to hold information in mind in the absence of sensory input, WM allows us to integrate events over time to guide adaptive, goal-oriented behavior and memories (Baddeley and Hitch 1974; Miyake and Shah 1999). As such, deficits in WM may have adverse effects for other cognitive domains, leading to deficits in effective decision making, planning, and long-term memory. A growing body of research suggests that selective attention is critical to support effective WM, by enabling selection and maintenance of relevant items in the face of competing distractors (Vogel and Machizawa 2004; Vogel et al. 2005; Gazzaley and Nobre 2012; Stokes and Nobre 2012). In addition, it has been shown that it is possible to orient attention after WM encoding to prioritize or update information being maintained in WM (Griffin and Nobre 2003; Landman et al. 2003).

WM functions decline with aging (Babcock and Salthouse 1990; Salthouse 1992, 1994; Rypma and D'Esposito 2000; Park et al. 2002; Chen et al. 2003; Iachini et al. 2009; Nagel et al. 2009; Werkle-Bergner et al. 2012; Peich et al. 2013), which may have important deleterious consequences for other cognitive functions. It is important, therefore, to understand which aspects of WM are compromised, and the extent to which top-down attentional control may be able to mitigate deficits. Several studies have demonstrated that healthy elderly adults experience significant declines in their ability to use selective attention to guide the encoding of relevant material and suppress irrelevant items during WM encoding (Gazzaley et al. 2005, 2008; Zanto et al. 2010; Gazzaley 2013; see also Hasher and Zacks 1988; Hasher et al. 1999). A number of previous investigations have tested for the effects of attention on WM in elderly adults, and have reported deficits in modulating expectation and encoding of stimuli for effective WM performance (Gazzaley et al. 2005, 2008; Fabiani et al. 2006; Jost et al. 2011; Sander et al. 2012; Peich et al. 2013). These studies leave open the important question of whether healthy aging also compromises the ability to exert flexible attentional control “after” encoding, to prioritize the maintenance or retrieval of certain elements over others.

Prior work has shown that the ability to orient attention within WM can be tested by presenting cues during the WM maintenance interval that provides retrospective information about which items are likely to be relevant to guide subsequent performance (“retro-cues”; Griffin and Nobre 2003). Retro-cues lead to reliable performance benefits in young adults (Griffin and Nobre 2003; Landman et al. 2003; Nobre 2004; Nobre et al. 2007; Sligte et al. 2008; Makovski 2012; Rerko and Oberauer 2013; Williams et al. 2013; Rerko et al. 2014). Preserved abilities to orient attention within WM would mean that, in contrast to preparatory attentional control during WM encoding, retrospective attentional control remains relatively intact in healthy aging.

In the current experiment, we tested whether elderly participants are able to exert flexible control over WM contents. We recruited a large sample of older adults to test for differences in the ability to orient attention to the contents of WM, and to investigate the neural correlates of spared versus impaired WM control. By capitalizing on variability within a homogenous cohort of older participants, we hoped to circumvent the inevitable extraneous nuisance variables that can contribute to comparisons of different age groups (motivation, fatigue, exposure to computer technology, medication, etc.). Furthermore, investigating individual differences within an elderly age group can tell us about the mechanisms that relate to successful aging. To our knowledge, 2 studies have tested the effectiveness of retro-cues in elderly participants (Duarte et al. 2013; Newsome et al. 2015), and both showed a significant impairment with healthy aging. We revisited this question by combining retro-cues with a WM precision task that enabled us to measure benefits in memory recall and in the quality of WM representations. We used a model-based analysis to explore whether putative retro-cue benefits arise from either an increased probability to retrieve relevant items or an increase in the precision of representations.

We recorded the neural activity during task performance using magnetoencephalography (MEG) to chart the temporal dynamics of oscillatory markers of orienting attention within WM. Similar to the effects observed when spatial attention is deployed in perceptual tasks (Worden et al. 2000; Thut et al. 2006; Rihs et al. 2007; Kelly et al. 2009; Gould et al. 2011), a robust marker for the deployment of spatial attention within WM is the systematic decrease in the power of alpha oscillations contralateral to the location of the cued WM item (Poch et al. 2014; Myers et al. 2015; Wallis et al. 2015). In contrast to the sustained desynchronization found after anticipatory attention shifts, modulations of alpha power during internal shifts of attention to items within WM appear to be more transient (Myers et al. 2015; Wallis et al. 2015). These rapid dynamics may reflect a transient process of changing excitability in—or access to—sensory cortex (Myers et al. 2015; Wallis et al. 2015).

We found that, as a group, elderly participants benefitted significantly from retro-cues. The behavioral benefits were mainly associated with an increased probability of retrieving the attended item and decreased guessing or confusion with other items. Neural markers of orienting attention in WM were similar to those described for younger populations (Poch et al. 2014; Myers et al. 2015; Wallis et al. 2015). At the individual level, the dynamics of the oscillatory markers were strongly predictive of performance benefits arising from flexible control over WM.

## Materials and Methods

### Participants

The study received ethical approval from the Central University Research Ethics Committee of the University of Oxford. All participants provided written informed consent and were compensated for their time and travel expenses.

Eighty-one healthy older adults (aged 60–87 years) were recruited from the community via local media and public advertisements. Of these, 75 participants were able to complete the current experiment. Reasons for withdrawing from the study included difficulty with traveling to the assessment center or instances of poor health. One further participant was unable to perform the task above chance level and therefore was excluded from the analysis (see Behavioral Data Analysis). The remaining 74 participants (42 females) were 60–87 years old (mean 68.8 ± 0.82 years), had 16 ± 0.47 years of education. All participants were fluent in English, had normal or corrected-to-normal vision and hearing, and scored >26 on the Mini-Mental State Examination (Folstein et al. 1975). None of the participants had any current diagnosed psychiatric or neurological disorder, and none were taking psychoactive medication.

Data from 61 participants were used in the MEG analysis [aged 60–87 years; mean 69.22 ± 0.92 standard error of the mean (SEM); 16.1 ± 0.52 years of education; 32 females]. Five participants were excluded because the structural magnetic resonance imaging scans revealed significant cortical atrophy. Seven further participants were excluded because the MEG data contained excessive artifacts and were consequently discarded before any processing (this included one participant who responded randomly in the task; see Behavioral Data Analysis). Data from one further participant were not saved due to a technical failure.

### WM Precision Retro-Cueing Task

**Figure 1.**
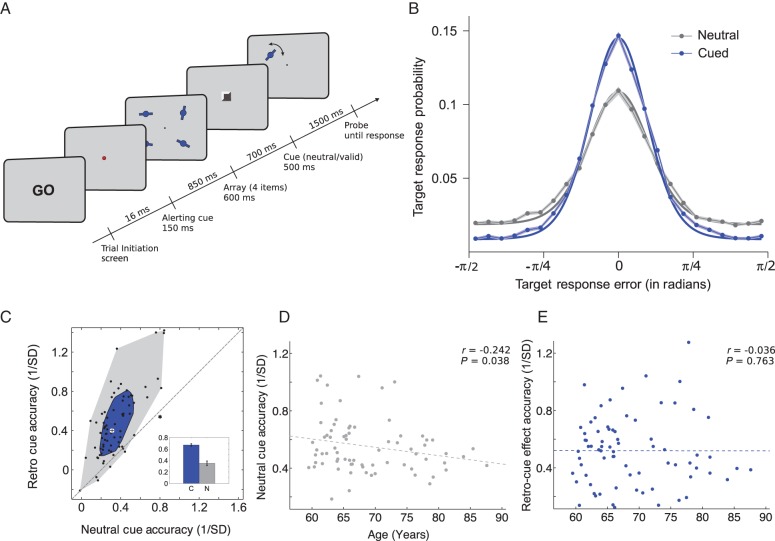
Task schematic and behavioral results for 74 participants. (*A*) Task schematic. Participants pressed a button to initiate each trial at the “GO” screen. Participants encoded 4 orientation stimuli into WM. After a fixed delay of 700 ms, a retro-cue appeared. In half the trials, a spatial retro-cue (100% valid) appeared, indicating the location of the item that would subsequently be probed. In the other trials, a neutral retro-cue provided no predictive information about the location to be probed. After another fixed delay of 1500 ms, a randomly oriented probe bar appeared in one of the quadrants. Participants indicated the orientation of the item at that location in the memory array by rotating the bar and confirmed with a button press. After responding, the correct orientation of the memory-array item was overlaid as feedback (not shown here). (*B*) Response error distributions centered on the target for spatial and neutral retro-cue conditions. The mean error is plotted at each bin and connected by the lines for the spatial (blue) and neutral (gray) retro-cue condition and the shading shows the SEM. The smooth lines show the model fit (weighted mixture of the von Mises and uniform distribution). *x*-Axis shows response error in radians, and *y*-axis is the response error probability. (*C*) Scatter and bag plot and bar plot showing the effect of the spatial retro-cue on WM behavioral measures. The small bar-plot inset shows WM accuracy for the spatial (blue) and neutral (gray) cue conditions (***P < 0.0001). For the scatter and bag plot, dots represent individual participants, *x*-axis is accuracy on neutral retro-cue conditions and the *y*-axis is accuracy on spatial retro-cue conditions. Dots above the diagonal represent participants who exhibited an increase in WM accuracy in the retro-cue conditions relative to the neutral-cue conditions and dots below the diagonal represent a decrease in accuracy in the retro-cue relative to neutral-cue conditions. The inner blue bag includes 50% of the data with the largest depth, the outer gray polygon contains all other nonoutlier data points, and the Xs outside of the shaded areas represent outliers. The cross at the center of the bag plot represents the center of mass of the bivariate distribution of empirical data (Rousseeuw et al. 1999). (*D*,*E*) Age was negatively correlated with accuracy in the neutral-cue condition (*D*) but not with retro-cue benefit (*E*). Correlation values are Spearman's rank correlation coefficients.

The main experimental task tested the number and quality of representations that individuals could maintain in WM, as well as their ability to orient attention within WM flexibly to prioritize relevant items. A WM precision task (Zhang and Luck 2008) was combined with a retro-cueing manipulation (Griffin and Nobre 2003). Figure 1*A* shows a schematic diagram of the task.

On each trial, participants encoded an array of 4 “orientation” items into WM, and were subsequently probed to recall one item after a delay. On spatial retro-cue trials, the location of the relevant item was indicated by a 100% predictive spatial cue during the delay interval. Spatial retro-cues appeared 700 ms after disappearance of the stimulus array. On neutral retro-cue trials, the cue provided no spatial information about the item to be probed.

Trials were self-initiated. A “GO” screen signaled that participants could initiate the trial by pressing a button on a MEG-compatible response pad. A red fixation point followed (150 ms in duration) alerting the participant of the upcoming stimulus array. The WM array appeared after 850 ms and remained visible for 600 ms. Four orientation stimuli were positioned in the 4 quadrants (centered at 4.8° horizontally and 4.8° vertically from fixation). Each array stimulus consisted of an oriented bar (2.32° in length and 0.16° in width), with a disc (0.72° diameter) at its center. Spatial or neutral retro-cues appeared after a delay of 700 ms. In half the trials, the cue indicated the location of the item that would subsequently be probed (100% validity). In the other trials, the cue provided no predictive information about the location to be probed. Cues were made up of a small black square (0.96° by 0.96°) presented centrally for 500 ms. In spatial retro-cue trials, 2 sides were colored white, forming an arrow pointing to one quadrant location. In neutral retro-cue trials, no sides were colored. After another 1500-ms delay, a probe bar appeared in one of the screen quadrants in a random orientation. Participants used the response pad to adjust the orientation of the probe stimulus so that it matched the orientation of the remembered item previously presented at that location. The pad contained 2 buttons; one button rotated the item clockwise and the other button counter-clockwise. Participants used their right hand to adjust the orientation, and then made a separate button-press response with the left hand to confirm their response. The maximum allowed recall time was 8500 ms. After the confirmatory button press, the actual orientation of the memory-array stimulus was overlaid over the participant's recall, providing feedback (200 ms duration). The intertrial interval from the feedback to the GO screen was 50 ms.

The task was programmed in Matlab v.7.10 (MathWorks) and presented using the Psychophysics Toolbox v.3.0 package (Brainard 1997). Stimuli were back-projected (Panasonic PT D7700E) onto a screen at a viewing distance of 120 cm with a spatial resolution of 1280 by 1024 pixels and a refresh rate of 60 Hz.

### Experimental Procedure

Participants completed a practice session in a “mock” MEG scanner before completing the experiment in the MEG scanner room. The practice session was used to familiarize the participant with the scanning environment and with the response demands of the task. The experimenter provided verbal instructions about the task using a slide show to illustrate the stimuli and experimental procedure. The “mock” scanner contained the same scanner layout, projector, and response pads as the MEG scanner room.

During the practice session, participants completed a visual matching task with oriented bars (2.88° length, 0.24° width, disc diameter 0.88°) to ensure participants understood the task and response method, and to verify that their visual acuity was sufficiently good to complete the task. In each trial, an oriented bar was presented at the top half and another at the bottom half of the screen. Participants had to adjust the orientation of the bottom bar to match the orientation of the bar above. All participants completed 24 trials. The stimuli were the same as those in the main experiment, except that the stimuli in the visual matching practice were larger.

Once participants were proficient at judging stimulus orientation and using the response pads to provide accurate responses, they completed practice trials of the main WM-precision task described above. In a first set of 24 trials, no cues were presented. Retro-cues were introduced in a second set of 24 trials. In this set, only valid spatial retro-cues were used. In a final set of 24 trials, both valid spatial retro-cues and neutral cues were intermixed randomly, as they would appear in the main task. This preparation procedure took approximately 20 min.

After setting up participants for MEG recordings, the main experimental task was completed in the MEG scanner. Participants completed 6 blocks of 40 trials, resulting in 240 trials (120 trials in the spatial and neutral retro-cue conditions). Spatial and neutral retro-cue trials were randomly intermixed within each block. Each spatial location was cued and probed with equal probability. Participants were asked to fixate on the center of the screen throughout each trial until the probe appeared. They were free to move their eyes while they adjusted the orientation of the probe stimulus and until they initiated the subsequent trial.

### Behavioral Data Analysis

The aim of the data analysis was to characterize the number of items older adults could maintain in WM, the precision of their representations, and their ability to orient attention to cued items in WM. We quantified various components that contribute to WM performance. Accuracy (reciprocal of the circular standard deviation of the recall error distribution; 1/*σ*) was calculated for each participant (Bays and Husain 2008), measuring the variability in the recall error. (As described in Bays et al. 2009, a correction was applied in which the standard deviation for circular data was taken subtracting the value expected by chance [values taken from a uniform distribution].) It is important to point out that this measure does not distinguish between types of errors such as inaccurate responses and random guesses. Therefore, to model different sources of error, a mixture model was applied (Zhang and Luck 2008; Bays et al. 2009), which attributes the distribution of recalls to a mixture of 3 separate components: the probability of responding to the target, responding to a nontarget, and responding at random (guessing). Orientations are assumed to be recalled with Gaussian variability. The model is described by the following equation:

(1) $$p(\hat \theta ) = a{\Phi _K}(\hat \theta - \theta ) + \beta \; \displaystyle{1 \over m}\; \mathop \sum \limits_i^m {\Phi _k}(\hat \theta - {\varphi _i}) + \gamma \; \displaystyle{1 \over {2\pi }}\; $$

where *θ* is the actual orientation of the target, $\hat \theta $ is the reported orientation, Φ*κ* is the von Mises distribution (circular analog of the Gaussian) describing recall variability with mean zero and precision parameter *κ* (precision). The probability of reporting the target is given by *α*, the probability of mistakenly reporting a nontarget is given by *β*, and *φ*_1_, *φ*_2_, *φ_m_* … are the orientations of the *m* nontarget items. The probability of responding randomly (guess rate) is given by *γ* = 1 – *α* − *β*. Maximum-likelihood estimates of each parameter (*α*, *β*, *γ*, and *κ*) were obtained for each participant and condition (spatial retro-cue, neutral retro-cue) by using an expectation maximization algorithm. To ensure that we found a global maximum for the model fit, the optimization procedure was repeated multiple times using various initial parameter values. These parameter estimates were compared between the spatial and neutral retro-cue conditions using paired *t*-tests.

Effect size (Cohen's *d*) was computed to test the magnitude of the retro-cue benefit (the difference between spatial and neutral retro-cue conditions) with the following formula:

(2) $$d = \displaystyle{{{\mu _2} - {\mu _1}} \over \sigma }$$

where *μ*_2_ and *μ*_1_ are the condition means and *σ* is the pooled standard deviation of the 2 conditions. Modeling and statistical analyses were conducted in Matlab R2013a, Matlab's Statistics Toolbox and IBM SPSS Statistics 21.

The Rayleigh's test was used to determine whether the response errors for each participant were distributed nonuniformly around a circle. One participant was removed from the analysis because the Rayleigh's test was nonsignificant (*P* = 0.12), for both spatial and neutral retro-cue conditions, reflecting a random distribution of responses.

Correlations with behavioral data were conducted by computing Spearman's rank correlation coefficients. Correlational analyses were performed between age and accuracy (1/*σ*) in the neutral retro-cue condition and retro-cue benefit (spatial retro-cue minus neutral retro-cue). Correlation coefficients were transformed into *z*-scores using Fisher's *r*-to-*z* transformation and compared with test for significant differences (Cohen and Cohen 1983).

### MEG Scan

MEG data were acquired using an Elekta Neuromag 306-channel system (204 planar gradiometers, 102 magnetometers) with a sampling rate of 1000 Hz. A band-pass filter of 0.03–330 Hz was applied during acquisition. Eye movements were monitored on-line with a MEG-compatible eye-tracker (EyeLink 1000, SR Research, Ottawa, ON, Canada) recording at 500 Hz. If participants broke fixation during trials, we reminded them to refrain from moving their eyes in the next break. The electrocardiogram and the vertical and horizontal electrooculograms (EOGs) were also recorded. Head position was monitored during the experiment with emitting coils affixed to the participant's head. The positions of these coils were digitized using a Polhemus 3D tracking system (Polhemus, EastTrach 3D). The Polhemus probe was used to obtain a set of ∼100 points to record the shape of the participant's head.

Each participant completed 6 task blocks with 40 trials each. These were collected during 2–4 successive MEG recording sessions lasting approximately 25 min each, depending on the duration of breaks and reaction times of individual participants.

### MEG Analysis

MEG data were analyzed using custom-written MATLAB scripts, the in-house OHBA Software Library (OSL), SPM8 (Litvak et al. 2011), and Fieldtrip (Oostenveld et al. 2011). The epoch of interest used for the analyses was the period between the retro-cue and the onset of the probe stimulus.

#### MEG Preprocessing

The continuous MEG data were visually inspected to remove channels with high levels of noise. Elekta's Maxfilter Signal Space Separation (SSS) algorithm was then applied to attenuate signals originating outside the head. The algorithm decomposes the data into a set of spherical harmonic basis functions and rejects components estimated to come from outside a sphere defined around the head. The data are then re-projected onto the MEG sensors. This final step also compensates for head movements by transforming the position of the interim representation relative to the sensors before re-projecting the data.

Continuous data were downsampled to 250 Hz and band-pass filtered between 1 and 100 Hz. These data were cut into 3.5-s epochs running from the onset of the WM array to the onset of the probe stimulus. Resulting epochs were visually inspected for artifacts. EOG traces were used to identify trials containing saccades. Trials with abnormal variance in the MEG signal or saccades during the WM maintenance period were excluded from subsequent analysis. Eyeblink components were detected over the whole continuous dataset and regressed out using independent component analysis. Only planar gradiometers were used for the MEG analysis. The total number of trials excluded from the MEG analysis was 594 of 7320 trials, or 8.11% (spatial retro-cue condition). On average, 10.2 ± 3.4 trials were excluded for each participant. The number of exclusions did not differ between trials with left (5.0 ± 1.7) and right (5.2 ± 1.7) retro-cues.

#### Time–Frequency Analysis

We computed a time–frequency representation (TFR) of power using a Fourier transform over sliding time windows in 40-ms steps. The width of the sliding time window was variable in duration: for each frequency, the window width was 4 cycles long. The time-domain signal was multiplied with a Hanning taper of equal length. Estimates were obtained at frequencies from 4 to 35 Hz in 1-Hz steps.

The power spectra for each cue condition were averaged over trials. The power time-series in the planar gradiometers were combined (Cartesian sum), resulting in a 102-channel combined planar gradiometer map of power in sensor space. For each participant, we contrasted the power spectra in the left minus the right spatial retro-cue conditions, normalized by the power of both conditions:

(3) $$\displaystyle{{\hbox{Left}} \over {\hbox{Left} + \hbox{right}}} - \displaystyle{{\hbox{Right}} \over {\hbox{Left} + \hbox{right}}}$$

Across the group, we tested for significant lateralization of brain activity according to spatial cues using paired *t*-tests and used spatial cluster permutation statistics to control for multiple comparisons. Sensor-space cluster permutation statistics were computed by permuting cue condition labels (left and right spatial retro-cue conditions) using Fieldtrip's ft_freqstatistics (10 000 iterations). Clusters were formed in space (sensors) and time, averaging over the alpha band (8–14 Hz) and the beta band (15–30 Hz) separately, and tested for significance against the permuted distribution. Control analyses were also performed in the theta band (4–7 Hz).

To verify that distinct alpha-band and beta-band peaks were observable in our elderly cohort, and that they conformed to the conventional frequency ranges used in our analyses, we plotted the spectral distribution of power over sensors showing maximal alpha and beta lateralization. A topographical analysis of variance (TANOVA, see Murray et al. 2008) was used to compare the topographies of alpha-band and beta-band lateralization in the period after the retro-cue. The difference between 2 topographies was computed by taking the square root of the sum of squared differences between conditions at each sensor, normalized by the variance across all sensors. This value was compared against a permutation distribution derived through computing values with randomly shuffled condition labels over 10 000 iterations.

#### Attentional Modulation Index and Correlating with Behavior

To explore the relationship between neural activity and the deployment of retrospective attention, we characterized the time course of alpha lateralization by computing an attentional modulation index (AMI) for each participant. To generate the AMI, we selected sensors involved in retrospective attention using a cluster-based analysis, and then subtracted average alpha power activity in the sensors ipsilateral to the attended hemifield (positive) from the sensors contralateral to the attended hemifield (negative), where a higher AMI meant more alpha lateralization and a lower AMI meant less alpha lateralization. To select sensors in an unbiased way, we used a leave-one-out method, and tested for differences between the normalized (as before) left and right cue conditions in the alpha band using paired *t*-tests and cluster permutation statistics during the delay period after the retro-cue (as above, but with 100 iterations) for 60 of 61 participants. We used the significant sensors to calculate the AMI for the left-out participant and repeated the procedure for all participants. For each left-out participant, one positive (left) and one negative (right) clusters were identified. Clusters were highly overlapping across participants. The same analysis was conducted in the beta band. We tested the significance of the AMI using one-sample *t*-tests over time points after cue offset and used cluster-based permutation testing (10 000 iterations) to correct for multiple comparisons across time, with a cluster-forming threshold (and a cluster mass significance threshold) of *P* < 0.05.

To compare the dynamics of neural effects in individuals with high versus low cueing benefit, we split the participants into 2 groups based on the size of the behavioral retro-cue benefit in each participant (spatial minus neutral retro-cue accuracy, 1/*σ*, median split). We confirmed that there were no differences in the numbers of excluded trials between the 2 groups (mean high-performers = 9.1 ± 5.1, mean low-performers = 10.0 ± 4.5; *t*_(56.8)_ = −0.14, *P* = 0.89). Initially, splitting the data led to a significant difference in age (independent samples *t*-test between groups: *t*_(49.8)_ = −2.43, *P* = 0.02). Therefore, we performed the median split on the residuals of a regression of age against performance (independent samples *t*-test between groups: *t*_(50.9)_ = −1.7, *P* = 0.1).

To compare the time course of lateralization between groups, we split the duration of significant alpha AMI at the group level into 3 bins, giving an early, middle and late lateralization period. Differences in groups for the early, middle, and late periods of alpha and beta modulation were tested using mixed ANCOVAs, regressing out the effect of age. Mauchly's test was used to test for sphericity of the data. For both ANCOVAs, the assumption of sphericity was violated; therefore, degrees of freedom were corrected using Huynh-Feldt estimates of sphericity, according to the recommendation to use the Huynh-Feldt correction when the epsilon parameter is >0.75 (Girden 1992).

To supplement the tertiles analysis and ensure that any difference in lateralization time course did not reflect the choice of arbitrary time points, we also performed a cluster-based analysis. A mixed one-way analysis of variance (ANOVA) tested for the effects of group (high retro-cue benefits, low retro-cue benefits), frequency (alpha AMI, beta AMI), and group × frequency interactions at each time point in the maintenance period after cue offset, and cluster-based permutation testing was used to control for multiple comparisons across time with a cluster-forming threshold of *P* < 0.05 and 10 000 iterations. To test for the effect of Group, group labels were randomly permuted, and the sum of the largest cluster of *F* values over time (with *P* < 0.05) was saved to build up a null distribution. The same procedure was performed to test for the effect of frequency, permuting the frequency labels but keeping group constant. Finally, we permuted both group and frequency and built up a null distribution to test for the interaction effect (Anderson and Ter Braak 2003). We compared the original results with the right side of the null distributions (one-tailed test) because only an effect that is significantly greater than the permutation distribution of *F* values would be interpretable.

## Results

### Behavioral Results

#### Main Experiment

As a group, the elderly participants were able to use spatial retro-cues to improve working-memory performance [accuracy for spatial retro-cue: 0.67 ± 0.04; neutral retro-cue 0.35 ± 0.02; *t*_(73)_ = 11.4, *P* = 7.24 × 10^−18^; effect size (Cohen's *d*): 1.17; Fig. 1*B*,*C*]. There was a significant negative correlation of accuracy on neutral retro-cue trials with age (*r* = −0.24, *P* = 0.038; Fig. 1*D*), but no relationship between the ability to use a spatial retro-cue to improve WM accuracy and age (spatial retro-cue accuracy minus neutral-cue accuracy: *r* = −0.036, *P* = 0.76; Fig. 1*E*). To compare the extent to which each effect correlated with age when any common variance in performance across the conditions was removed, we repeated the analyses using partial correlations. Accuracy on neutral trials still showed a negative correlation with age when partialling out the effects of retro-cueing (*r* = −0.21, *P* = 0.006), and retro-cueing benefits showed no relation to age when partialling out the performance on neutral-cue trials (*r* = 0.003, *P* = 0.98). When compared directly using Fisher's *r*-to-*z* transform, these correlation coefficients differed significantly (*z* = 2.00, *P* = 0.046).

The pattern of behavioral results was not significantly affected by whether participants were taking calcium-channel blockers (15 of 74 participants) or had a previous history of depression (11 of 74 participants).

**Figure 2.**
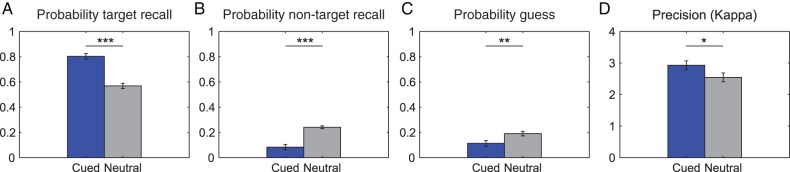
Significant retro-cue benefits in WM components from the mixture-model analysis for 74 participants. The probability of target recall (*A*) and precision (*D*) were significantly higher in the retro-cue relative to the neutral-cue condition, and the probability of nontarget (*B*) and guess responses (*C*) were significantly lower in the retro-cue relative to the neutral-cue condition. ***P < 0.00001; **P < 0.01; *P < 0.05.

The mixture-model analysis revealed that this effect was attributable to an increase in the probability of reporting a target (*t*_(73)_ = 10.1, *P* = 1.78 × 10^−15^; effect size: 1.3; Fig. 2*A*), a decreased probability of mistakenly reporting a nontarget (*t*_(73)_ = −6.85, *P* = 1.93 × 10^−9^; effect size: −1.64; Fig. 2*B*), and a decrease in guess rate (*t*_(73)_ = −2.78, *P* = 0.007; effect size: 0.5; Fig. 2*C*). The measure of precision in the mixture model (concentration parameter *κ*) was also significantly modulated by a retro-cue, though the effect size was small (*t*_(73)_ = 2.18, *P*= 0.032; effect size: 0.32; Fig. 2*D*). The 61 participants who were submitted to the MEG analysis showed a similar pattern of behavioral performance except that precision was no longer significantly modulated by the cue. Statistical values for the subset of participants were: accuracy for spatial retro-cue (0.68 ± 0.04; neutral retro-cue 0.35 ± 0.02; *t*_(60)_ = 11.4, *P* = 1.34 × 10^−15^; effect size:1.24), probability for target (*t*_(60)_ = 10.1, *P* = 4.4 × 10^−15^; effect size: 2.00), probability for nontarget (*t*_(60)_ = −6.85, *P* = 1.7 × 10^−7^; effect size: −1.52), guess rate (*t*_(60)_ = −2.78, *P* = 7.0 × 10^−4^; effect size: −1.07), precision (*t*_(60)_ = 1.11, *P* = 0.27; effect size: 0.17).

### MEG Results

#### Alpha Power Lateralization During Retroactive Attention

**Figure 3.**
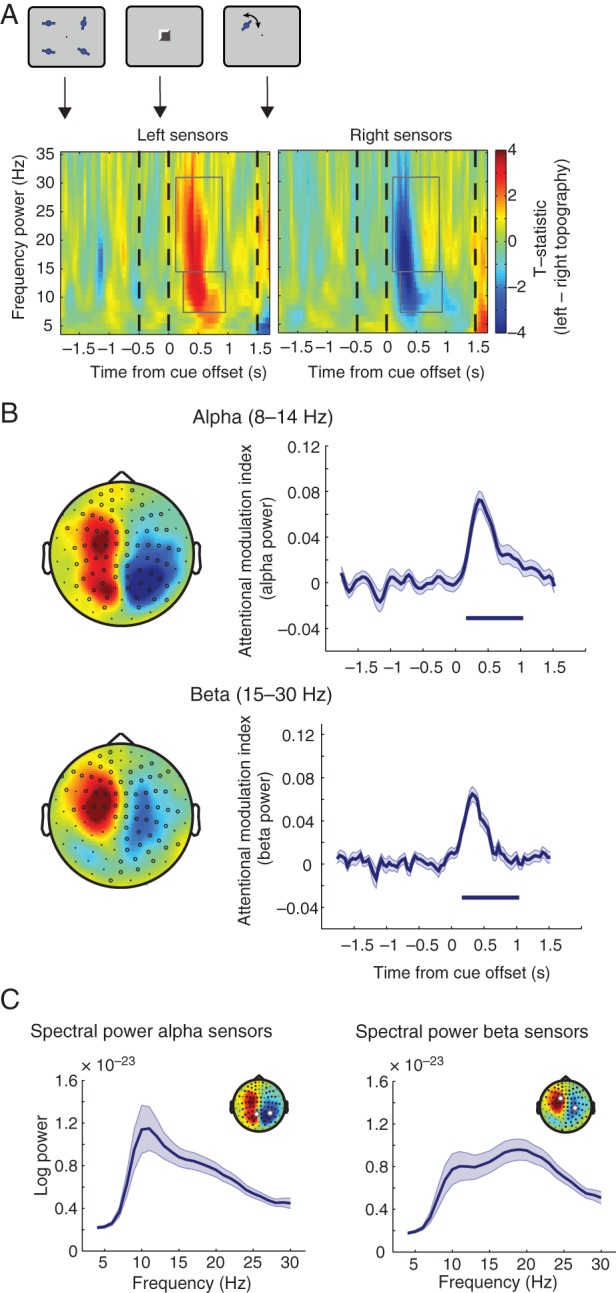
Alpha and beta lateralization. (*A*) Time course of alpha (8–14 Hz) and beta (15–30 Hz) power lateralization from sensors for the left minus right retro-cue contrast. Left: Increase of event-related synchronization (ERS) of alpha/beta power on the ipsilateral hemisphere to the attended side following a retro-cue. Right: Decrease of ERS of alpha/beta power on the contralateral hemisphere to the attended side following a retro-cue. Gray boxes denote significant increase/decrease in lateralization for alpha and beta bands. *x*-Axis is time from cue offset; *y*-axis is frequency power. (*B*) Left: Topography of alpha (top) and beta (bottom) power for the left minus right retro-cue contrast at 120–800 ms (alpha) and 8–840 ms (beta) after cue offset. Right: Time course of alpha (top) and beta (bottom) attention modulation index (AMI). The AMI is the average of the ipsilateral sensors minus average of the contralateral sensors' alpha or beta power, respectively (sensor selection via a leave-one-out method; see Materials and Methods). *x*-Axis is time from cue offset; *y*-axis is AMI. The bar below the AMI denotes significant lateralization compared with zero (both alpha and beta AMI: *P* < 1.0 × 10^−4^ cluster-corrected over time). (*C*) Spectral power for peak alpha and beta lateralization sensors. Mean log power for each frequency from 4 to 30 Hz in 1 Hz steps at sensors that exhibited peak modulation during the period of significant alpha lateralization (120–800 ms; left) and beta lateralization (8–840 ms; right) averaged over left and right retro-cue conditions, showing separate alpha and beta spectral peaks.

Retroactive attention to spatial locations in WM elicits a pattern of lateralized alpha power activity in posterior cortex in younger adults in both MEG (Poch et al. 2014; Wallis et al. 2015) and EEG (Myers et al. 2015). Here we tested for an alpha lateralization effect related to retroactive attention in older adults. We performed a sensor-space analysis comparing alpha-band power (8–14 Hz) after left retro-cues versus right retro-cues. Performing a cluster permutation test revealed a significant negative cluster (Fig. 3*A*,*B*, top panel) over right sensors from 200 to 800 ms after cue offset (*P*= 7.0 × 10^−4^) and a significant positive cluster over left sensors from 240 ms to 800 after cue offset (*P* = 6.0 × 10^−4^). We noted that the distribution of the alpha effects in the left hemisphere extended more anteriorly than is typically reported, including central and frontal sensors (cf. Foxe et al. 2014).

We performed the same analysis on the beta band (15–30 Hz) and found a significant negative cluster over right sensors from 8 to 440 ms after cue offset (*P* < 1.0 × 10^−4^) and a significant positive cluster over left sensors from 120 to 840 ms after cue offset (*P* = 4.0 × 10^−4^) (Fig. 3*A*,*B*, bottom panel). We also performed the same analysis on the theta band (4–7 Hz) and found no significant effects.

Plots of the raw spectral power at the sensors with maximal alpha-band and beta-band AMI (Fig. 3*C*) confirmed that their peak distributions were similar to the conventional frequency ranges used for analysis. Power in the alpha band peaked at 11 Hz over lateral posterior sensors, while power in the beta band peaked at 19 Hz over lateral central sensors. A comparison of the topographies associated with alpha-band versus beta-band lateralization using a TANOVA (Murray et al. 2008) in the period after the retro-cue (8–800 ms) showed them to be significantly different (*P* < 1.0 × 10^−4^). A similar analysis confined to the periods of strongest alpha lateralization (240–800 ms) and beta lateralization (120–440 ms) found similar results (*P* < 1.0 × 10^−4^).

#### Temporal Dynamics of Alpha Lateralization Predict WM Performance

**Figure 4.**
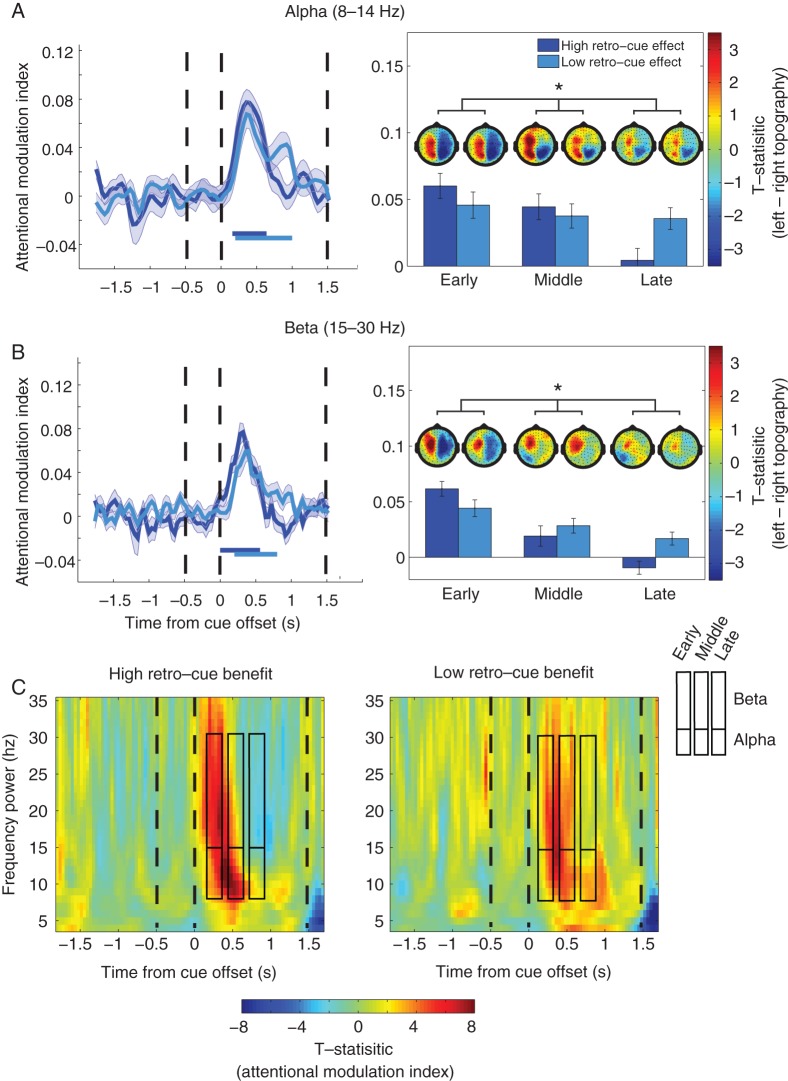
Temporal profile of alpha/beta lateralization reflects magnitude of retro-cue benefit in WM accuracy. (*A*) Left: Time course of the attentional modulation index (AMI) for high and low cueing benefit groups in the alpha band. The high cueing group exhibited a large increase of AMI after cue offset that shortly went back to baseline, whereas the low cueing group had a numerically smaller increase of AMI that lasted for longer. Right: Bar plots showing a significant interaction between groups for the AMI in the early, middle and late periods. Topographies display *t*-stat images of alpha power lateralization for each group and averaged over the early, middle, and late time points above each respective bar plot. Topography color scale is kept constant with *t*-values of −4 to 4. The bars below the AMI denote significant lateralization (AMI significantly different from zero) in each group. (*B*) The same analysis is presented for beta. *Significant interaction (*P* < 0.05). (*C*) Time–frequency plots of the AMI in participants with high retro-cue benefits (left) and low retro-cue benefits (right) for visualization. *x*-Axis is time from cue offset; *y*-axis is frequency power.

To test what aspects of neural activity predicted the ability to benefit from spatial cues, we performed a median split based on the behavioral retro-cue benefit (controlled for age) for WM accuracy, and then contrasted alpha lateralization between groups using the AMI (see Materials and Methods). We were particularly interested in whether the strength and timing of the alpha AMI predicted performance. At the group level, alpha lateralization reflected in the AMI was statistically significant from 160 to 1000 ms after cue offset (*P* < 1.0 × 10^−4^). To characterize the time course of lateralization between groups, we split the duration of significant alpha AMI at the group level into 3 equal bins to form an early (160–360 ms), middle (480–680 ms), and late (800–1000 ms after cue offset) lateralization period, leaving out the 2 time points between each time period. A mixed ANCOVA with within-subject factor time bin (early, middle and late), between-subject factor group (High and Low retro-cue benefit), and age as a covariate was used to test the amount of alpha AMI over the early, middle, and late time periods between groups (Fig. 4*A*). This revealed a significant interaction (*F*_1.77,100.6_ = 4.47, *P* = 0.017, *ηp*^2^ = 0.73), but no main effect of time bin (*F*_1.77,100.6_ = 1.53, *P*= 0.3, *ηp*^2^ = 0.03) or group (*F*_1,57_ = 0.314, *P* = 0.58, *ηp*^2^ = 0.005). The interaction was due to significantly less degree of AMI in the high relative to low performers in the late epoch (*t*_(57.8)_ = −2.79, *P* = 0.007, effect size: −0.72), but not in the early (*t*_(58)_ = 1.06, *P* = 0.3, effect size: 0.27) or the middle (*t*_(57.7)_ = 0.8, *P* = 0.43, effect size: 0.21) epoch.

We conducted the same analysis on the beta AMI to explore whether the group differences were exclusive to the alpha band (Fig. 4*B*). A mixed ANCOVA with within-subject factor time (early, middle, and late) and between-subject factor group (high and low retro-cue benefit) was conducted on the beta AMI using the same time periods for the alpha AMI analysis, controlling for age. This also revealed a significant interaction (*F*_1.9,101.4_ = 6.3, *P* = 0.002, *ηp*^2^= 0.108), but no main effect of time bin (*F*_1.9,101.4_ = 1.15, *P* = 0.85, *ηp*^2^ = 0.005) or group (*F*_1,57_ = 1.45, *P* = 0.23, *ηp*^2^ = 0.025). Independent sample *t*-tests revealed that the interaction was due to a significant difference between the high and low performers in the late epoch (*t*_(58)_ = −3.27, *P* = 0.002, effect size: −0.85), with a trend in the Early epoch (*t*_(57.2)_ = 2.00, *P* = 0.051, effect size: 0.52) but not in the middle (*t*_(52.6)_ = −0.78, *P* = 0.43, effect size: 0.20) epoch.

To make sure our effects were not due to the arbitrary time periods selected for these analyses, we also conducted an analysis of variance using a cluster-based permutation approach. The analysis revealed a significant cluster distinguishing high and low performers between 800 and 1000 ms after cue offset, suggesting that modulation of alpha and beta power was longer lasting in the low-performance group (*P* = 0.026, one-tailed, cluster-corrected over time). There was a trend for a main effect of frequency between 400 and 680 ms (*P* = 0.07, cluster-corrected over time), suggesting that the AMI tended to be stronger for the alpha band than the beta band. There were no interactions, suggesting a similar type of modulation by performance group in the 2 frequency bands (*P* > 0.51, cluster-corrected over time).

## Discussion

We tested a large sample of elderly participants on a WM precision task with retro-cues and found that the ability to orient attention in WM is preserved in aging. Despite finding a reduction in baseline WM performance with increasing age, the cueing benefit was unaffected. Posterior alpha-band power was modulated after spatial retro-cues, suggesting that the oscillatory mechanisms that accompany retrospective attention (Poch et al. 2014; Myers et al. 2015; Wallis et al. 2015) are also retained in aging. The temporal profile of alpha-band lateralization corresponded with behavior: Participants with strong cueing benefits exhibited a short-lived, transient lateralization of alpha activity, whereas those with limited benefits showed a prolonged period of alpha lateralization. Finally, we found that these effects were also present in the beta band, which has not been reported previously in studies in young adults (Poch et al. 2014; Myers et al. 2015; Wallis et al. 2015).

In line with previous research on younger adults (e.g., Griffin and Nobre 2003; Landman et al. 2003; Nobre 2004; Nobre et al. 2007; Sligte et al. 2008; Makovski 2012; Rerko and Oberauer 2013; Williams et al. 2013; Rerko et al. 2014), healthy older adults were able to use retro-cues to benefit their WM performance. A model-based analysis showed that, as a group, there was a robust increase in the probability for reporting the target item and reduction in misreporting nontarget items. There was also a modest increase in the precision of the reported memorandum, although this effect was not significant in the reduced dataset (*N* = 61) used for the MEG analysis. In addition, we found that flexible orienting within WM did not decline with age, whereas baseline WM performance did. Specifically, WM accuracy in the neutral-cue condition decreased as a function of increasing age, replicating the commonly reported age-related decline in WM maintenance functions (e.g., Salthouse 1992, 1994; Reuter-Lorenz and Sylvester 2005; Iachini et al. 2009; Werkle-Bergner et al. 2012; Peich et al. 2013). In contrast, there was no correlation between the retro-cue benefit and age. Consistent with the current results, previous perceptual studies have shown that older adults can use spatial pre-cues to improve performance in perceptual target detection and discrimination tasks (e.g., Nissen and Corkin 1985; Tales et al. 2002; Madden 2007). However, 2 studies reported that older adults have an impaired ability to orient attention within WM using retro-cues (Duarte et al. 2013; Newsome et al. 2015; see below for further discussion). In addition to showing preserved orienting within WM in healthy aging, we found that these intact orienting functions may reduce the detrimental effect of age on WM performance.

Flexible orienting of attention within WM elicited a transient pattern of lateralized alpha activity in older adults, similar to what has been observed for young participants (Poch et al. 2014; Myers et al. 2015; Wallis et al. 2015), thereby indicating relative preservation of oscillatory mechanisms in healthy aging. This transient activity could reflect a shift of attention to the cued item in WM. For example, attentional selection could lead to a change in the item's representational state so that it can guide subsequent memory recall (Larocque et al. 2014; Zokaei et al. 2014). Following completion of the selection process, alpha lateralization may no longer be required (Wallis et al. 2015). This brief activation may represent a state of rapid memory access to the behaviorally relevant item within WM.

High variability between subjects in the strength and timing of alpha lateralization allowed us to investigate the neural markers of good versus poor orienting ability. We found that participants with high and low retro-cue benefit had similar overall magnitudes of alpha lateralization in the delay after the retro-cue. However, the temporal profiles of alpha lateralization over the delay period differed markedly between groups. Participants with a high retro-cue benefit had a strong increase of alpha lateralization immediately after the cue, which quickly returned to baseline. In contrast, participants with a low retro-cue benefit had a moderate increase of alpha lateralization after the cue, which was sustained for a longer period of time before it went back to baseline. If alpha lateralization after a retro-cue in fact corresponds to a punctate process of retrieval, or a change in accessibility, then lateralization should last no longer than this retrieval or transition process itself. We speculate that the more sustained alpha lateralization in the low-performing group may reflect a less efficient memory selection process.

In our group of older adults, effects of attentional modulation were also present in the beta band. We ruled out that the effects observed in the beta band resulted merely from shifts in spectral power as a function of aging. In our cohort, alpha and beta bands had distinct peak frequencies and topographical distributions. These effects in the beta band have not been reported in similar studies testing younger participants (Myers et al. 2015; Wallis et al. 2015). Our findings are in line with previous EEG studies reporting greater beta power modulation in older adults relative to younger adults in attention and WM tasks over central electrodes (Karrasch et al. 2004; Deiber et al. 2010; Gola et al. 2013), although they have not specifically reported lateralized effects related to spatial attention.

The contribution of the beta-band lateralization during WM control in our task remains unclear. We speculate that older adults may employ additional strategies and neural resources to compensate for normal declines in aging (Cabeza et al. 2002; Grady 2008; Park and Reuter-Lorenz 2009), which may be reflected in beta-band modulation. For example, some studies report that older adults additionally recruit the motor network for cognitive and motor tasks (Rowe et al. 2006; Kopp et al. 2011; Deiber et al. 2013). However, because the effects of alpha and beta were not entirely separable in this study, future studies could use a longitudinal design to examine whether there are in fact separable effects and whether recruitment in the beta band is a consequence of aging.

Our study points to larger flexibility and executive control in older adults compared with other studies. In particular, our current behavioral findings stand in contrast to 2 previous studies showing that older adults were unable to benefit from retro-cues (Duarte et al. 2013; Newsome et al. 2015). We used a WM precision task with orientations, whereas both previous studies used a traditional color change-detection task. Our precision task may simply have been more sensitive to subtle behavioral differences between cue conditions (Zokaei et al. 2015). Furthermore, we used an accuracy measure that considered each participant's response variability (i.e., their inverse precision), whereas the previous study took standard reaction-time and accuracy measures. Another major difference between the studies was in the sample size (74 here compared with 18 and 19 in the previous studies). The absence of an effect may have been due to higher intersubject variability in behavioral performance in older adults (Botwinick 1978; Krauss 1980; Welford 1985), meaning larger samples may be required to observe reliable effects.

Prior studies have found that older adults are impaired in the ability to suppress irrelevant items from entering WM (Gazzaley et al. 2005, 2008; Zanto et al. 2010; Gazzaley 2013). These findings may seem at odds with the current results. One major difference is that these previous studies tested selective gating of input into WM, that is, the ability to suppress irrelevant items at encoding. Conversely, we tested for control over WM items after encoding. One purpose of the current study was to investigate whether older adults have deficits in exerting control over WM. We found that older adults had preserved abilities for orienting attention within WM, which suggests no deficit in the suppression of irrelevant items in WM. It is important to note that some studies that reported attentional deficits for the contrast between old and young groups also found differences within the old group, in which high performers show some suppression of irrelevant stimuli and low performers show no suppression (Gazzaley et al. 2005; Chadick et al. 2014).

A separate line of research investigating selective attention in aging suggests that older adults can use pre-cues to improve target detection and discrimination performance to the same degree as young adults (Nissen and Corkin 1985; Tales et al. 2002; Madden 2007), contrary to studies by Gazzaley and colleagues. However, in studies that reported age-related attention deficits, distractors were often salient and foveal (such as faces and houses), whereas research using pre-cues as well as the current study used small, peripheral items. These discrepancies might be related to the greater difficulty for suppressing salient, foveal distractors compared with peripheral distractors (Zanto and Gazzaley 2014). It is unclear whether our current results would still hold if irrelevant items were more salient or distracting. Future research should study whether control mechanisms within WM are impaired in aging when more salient distractors are combined with retro-cues.

While the present study showed that older adults, as a group, are able to orient their attention flexibly within WM to optimize performance, it was also able to demonstrate high individual variability in cognitive performance. This variability was linked to differences in the temporal dynamics of neural oscillations, which may be a marker for healthy attentional mechanisms in aging. Studying variability within elderly adults might be able to tell us about behavioral and neural signals that underlie successful cognitive aging. Indeed, to examine this question, one research group has already begun to study “superagers,” which are elderly adults who have unusually high memory abilities (Rogalski et al. 2013; Gefen et al. 2014, 2015; also see Nyberg et al. 2012). The current results show that different patterns of neural activity can distinguish between persons with different levels of flexible control in WM. This individual-differences approach may be a promising way to investigate the behavioral and neural markers of preserved cognition in aging.

## Funding

The research was funded by the National Institute for Health Research (NIHR) Oxford Biomedical Research Centre based at Oxford University Hospitals NHS Trust and University of Oxford, and by a Wellcome Trust Senior Investigator Award (104571/Z/14/Z, A.C.N.). Funding to pay the Open Access publication charges for this article was provided by the Wellcome Trust.
